# Applying Rasch analysis in refinement and validation of interpersonal skills measure for gifted children

**DOI:** 10.3389/fpsyg.2023.1236640

**Published:** 2023-08-31

**Authors:** Jie Yang, Zulhumar Reheman, Yunjie Liu, Sijie Zhao

**Affiliations:** ^1^Center for Experimental Economics in Education, Shaanxi Normal University, Xi’an, Shaanxi, China; ^2^Judith Herb College of Education, University of Toledo, Toledo, OH, United States; ^3^College of Physics and Information Technology, Shaanxi Normal University, Xi’an, Shaanxi, China

**Keywords:** child development, interpersonal skills, gifted children, measure validation, Rasch analysis

## Abstract

**Background:**

Interpersonal characteristics of gifted adolescents is important because of the potential influence on individuals’ psychological health and future professional success. Understanding the interpersonal characteristics requires valid and reliable measures. This study attempted to explicitly describe the application of Rasch analysis in the validation and development process of an existing measure of interpersonal skills among gifted adolescent in Ohio.

**Methods:**

We extensively evaluated the psychometric properties of the 40-item scale measuring the interpersonal competence of adolescents among the gifted population in Ohio (N = 127) using Rasch analysis. Multiple aspects of reliability and validity were tested including dimensionality, rating scale functioning, and fit statistics.

**Results:**

The internal consistency reliability of the scale was confirmed with an adequate fit to the Rasch model. However, the scale demonstrates relatively poor performance in terms of unidimensionality with our sample. Also, the rating scale categories were confusing given that our sample could not effectively distinguish some adjacent categories. Corresponding refinements have been made and the revised scale formed a meaningful linear progression with improved performance on unidimensionality, rating scale functioning, and fit statistics.

**Conclusion:**

The study provided evidence that the construct of interpersonal skills is measurable. Based upon the original Interpersonal Competence Questionnaires consists of 40 items, the author extracted and piloted a refined measure consisting of 31 items that performed a meaningful, theoretically consistent linear progression measure that could be used to measure the level of interpersonal skills of gifted children.

## 1. Background

A growing number of researchers have observed that noncognitive traits such as self-efficacy, personal motivation, and interpersonal skills are as important as cognitive abilities (Heckman et al., 2006; Cunha and Heckman, 2007) in the development of well-being in adulthood (Fredrickson, 1998; Peterson and Seligman, 2004; Segrin and Taylor, 2007). Interpersonal skills, which are one of the most important noncognitive skills, refer to the ability to establish healthy relationships with other people (Fredrickson, 1998). Because positive emotions and strong interpersonal skills play a critical role in the maintenance of psychological health (Fredrickson, 1998; Peterson and Seligman, 2004; Segrin and Taylor, 2007), children who have high levels of interpersonal skills tend to be more successful in their social relationships and derive greater pleasure from their activities as compared to their less skilled counterparts (Segrin and Taylor, 2007). Conversely, those who have poor interpersonal skills are at a greater risk for mental (e.g., depression) and physical health problems (Segrin and Taylor, 2007). Research has also shown that advanced interpersonal skills during the early years are related to desirable academic and professional outcomes in the later years (Jones et al., 2015), such as a greater likelihood of graduating from college and being employed, and a lower likelihood of committing crimes (Jones et al., 2015).

In the field of gifted education, researchers and educators have paid much attention to the social and emotional needs of gifted children (Fredrickson, 1998; Peterson and Seligman, 2004; Sapmaz, 2006; Segrin and Taylor, 2007). Studies have shown that highly capable gifted students experience more intense social and emotional struggles than their nongifted counterparts (Coleman and Cross, 1988; Brown and Steinberg, 1990). Specifically, gifted students are more likely to report difficulties in creating and maintaining friendships, experience feelings of social isolation (Cross et al., 1991, 1993), and exhibit perfectionistic tendencies (Schuler, 2000). These researchers also found that gifted adolescents reported such social problems more frequently than students who belonged to other age groups (Cross et al., 1991, 1993). However, there are also some different opinions regarding gifted children’s interpersonal skills. As Yoo (2016) found that scientifically gifted have higher interpersonal skills than general students accompanied with higher sense of humor and higher self-leadership; Gomes-Perez’s study also identified better abilities of interpersonal skills among children with high IQ than children with average IQ (Mar Gómez-Pérez and Dolores Calero, 2023). Studies also found that gifted children may have strengths in some dimensions of interpersonal skills such as understanding, collecting information, and resolving conflicts, but weaknesses in other dimensions such as social adaptation (Shechtman and Silektor, 2012).

The inconsistency in findings might be attributed to the study design and domains of the giftedness of the sample, but the very first step of understanding patterns and characteristics of gifted children’s interpersonal skills is to have reliable and valid measures of such construct. One of the most widely used measures is the Interpersonal Competence Questionnaire developed by Buhrmester et al. (1988). This five-point rating scale assesses five domains of interpersonal skills: (a) initiating relationships, (b) disclosing personal information, (c) asserting displeasure with others, (d) providing emotional support and advice, and (e) managing interpersonal conflict. Respondents are required to indicate their level of comfort in handling each situation using a 5-point scale. Reliability and validity evidence was approached based on Classical Test Theory in numerous studies. For example, Lee et al. (2012) demonstrated the domains and summary scores of ICQ to have a satisfactory internal consistency, producing a Cronbach alpha ranged from 0.81 to 0.90 on the subscales, and Cronbach’s alpha coefficients involving the total items of the scale was 0.93 for preadolescents and 0.92 for adolescents. Other studies further established the convergent validity of the ICQ (Buhrmester et al., 1988; Costa and McCrae, 1992; Riemann and Allgöwer, 1993; Kanning, 2006).

However, the above-mentioned reliability and validity estimates based on Classical Test Theory (CTT) may not be enough when used with a wider population. Modern Items Response Theory (IRT) can work as a complement to CTT as it provides unique information about scales. As a special case of one parameter logistic model, instrument validation using the Rasch model, which was originally developed by Georg Rasch for the investigation of reading ability in 1952, extends beyond CTT’s reliance on raw scores by estimating the extent to which participant responses fit a unidimensional linear pattern, guided by a theory of the construct. According to Bond and Fox (2015), the assumption that Rasch analysis is based on is the probability of a person passing a single item in a test is related to the assertions about a person’s ability and the difficulty of the item toward which the empirical data are tested. Moreover, the Rasch model enables researchers to understand fit-to-response patterns by putting data into a probabilistic framework. It builds the connection between persons and items and makes inferences about other samples of children and other samples of items. Empirical results from Rasch analyzes would therefore inform theory Bond and Fox (2015) by investigating the patterns in the data. These patterns can then be interpreted as evidence to support the present theory, or to refine the theory, and to provide guidance for further instrument development and analysis.

The purpose of the study was therefore to evaluate the extent to which the ICQ scale can be used to quantify the interpersonal skills of a sample of gifted children, so as to validate a psychometrically defensible measure for its use among the gifted population. The research questions for testing the reliability and validity of such a measure are listed below:

1. Reliability: do the items classify respondents into two or more groups that statistically differ in their levels of interpersonal skills? (This evidence is provided by Rasch diagnostics of person reliability and strata statistics).
2. Validity: is the measure sufficiently unidimensional? (This evidence is provided by Rasch principal components analysis [PCA] and fit statistics).
3. Validity: to what extent are the 5 response categories meaningfully distinguished by the respondents? (This evidence is provided by Rasch rating scale diagnostics).
4. Validity: do the items yield a stable and meaningful linear progression of the responses, corresponding to the theoretical framework of the construct? (This evidence is provided by interpretation of the Rasch Item-Person Map including targeting of person and item).

## 2. Methods

### 2.1. Participants

The target population of this study is adolescents who were officially identified as gifted and talented in at least one ability domain [i.e., intelligence, creativity, and socio-affective and sensorimotor abilities; Gagne, 2004] regardless of ethnicity in the state of Ohio. Gifted and talented students were excluded from the study if they did not undergo the standard process of the identification of giftedness according to specific state requirements. In Ohio, the identification of gifted children entails a process of referrals and whole-grade screening. That is, parents, teachers, and peers can refer the students for the identification and evaluation of giftedness. Districts must then provide whole-grade screenings in the domains of superior cognitive ability, specific academic abilities regarding reading, writing, mathematics, and creative thinking ability. The results of these assessments are used for the identification of giftedness.

Data collection occurred from April to August 2018. The questionnaire used for this study included basic demographic information of the participants and Buhrmester & Furman’s original scale of the ICQ questionnaire developed in 1988 (Buhrmester et al., 1988, see Supplementary File S1). Two separate rounds of sampling procedures were conducted to meet the minimum sample size required to ensure the robustness of Rasch analysis results (Chen et al., 2014). In the first round, 175 students were randomly selected from a list of students enrolled in gifted programs in local school districts of city A in Ohio. Parental consent forms and child assent forms were obtained from all participants. Fifty-nine students between the ages of 9 and 14 years (mean = 12.59; SD = 1.15), and their parents signed the consent forms, completed the surveys, and returned them to the researcher. Since Chen et al. (2014) concluded that Rasch analysis based on small samples (<50) identified a greater number of items with incorrectly ordered parameters than larger samples (>100), to ensure the robustness of the results, we attempted a second round of data collection following the same procedure. The final sample consists of 127 students with an average age of 12.59 (SD =1.15; range: 9–14). The majority of the students were Caucasian (82.7%), others were African American (12.5%), Hispanic (3.9), and Asian American (1%).

### 2.2. Construct theory of the ICQ

The construct theory of the ICQ hypothesized the potential structure of interpersonal skills reflected by a hierarchy of the ICQ items (see Appendix). The hierarchy helps to hypothesize which items manifest high interpersonal skills and which manifest low interpersonal skills. Based on a review of the existing literature on interpersonal competence, the hierarchy was designed to entail items that range from the most difficult (top) to endorse to the easiest (bottom). This means items located on top of the hierarchy can be endorsed more often for students with higher interpersonal competence.

The ICQ items are then conceptualized as a representation of three levels of interpersonal ability. Specifically, people who have advanced interpersonal abilities may be good at interacting with people whom they do not know, and sharing with them their inner experiences, especially the vulnerable and sensitive aspects of themselves. People who demonstrate high levels of interpersonal competence can also effectively manage conflict, and they have the courage to speak out about the things that make them unhappy. However, people who have low levels of interpersonal competencies typically find it difficult to endorse the items that measure these abilities (i.e., conflict management, and negative assertion). Instead, the items that measure the ability to provide emotional support, show empathic concern, and be a sensitive listener are relatively easier to endorse. Accordingly, even individuals with very low levels of interpersonal competence should be able to demonstrate some of these characteristics.

### 2.3. Data analysis

The ICQ data were analyzed using the Rasch Rating Scale Model (RSM) (Andrich, 1978), with WINSTEPS software (Linacre and Wright, 2004). Reliability estimates for the Rasch RSM included both the reliability coefficient (i.e., internal consistency) and separation statistics, the latter of which estimated the extent to which there are enough items of varying difficulty and persons of varying ability to construct a measure (Costa and McCrae, 1992). The first research question was thus examined in part by using the separation reliability, which was then transformed into a strata index [Strata = (4G + 1)/3; Wright and Masters, 1982], to indicate the number of statistically distinct groups of persons and items that could be differentiated on the measure.

To assess unidimensionality (second research question), Rasch principal component analysis (PCA) of the residuals was conducted to test whether a substantial amount of the systematic variance that is unrelated to the original linear measure exists in the data. Linacre (1999) suggested that a Rasch measure that accounts for ≥40% of the variance is indicative of a strong measurement dimension. In addition to the PCA, we also look at Rasch fit statistics to assess the extent to which each person and each item performed as expected. In other words, children with higher level of interpersonal competence should more strongly endorse the items than children with less interpersonal competence; items that indicate more interpersonal competence should be more difficult to endorse than items that indicate less interpersonal competence.

Answering the third research question entailed the examination of **threshold statistics for rating scale items to** examine the extent to which the rating scale is reliably used by the respondents, that is, if the categories were perceived as psychologically distinct and used by respondents in a consistent manner. Evidence of rating scale functioning is provided by the Andrich threshold value, which should increase across each level of the scale. The average measure is another statistic that should also increase in size as the level of the scale increases so that each rating scale category specifies a distinct meaning along the measured variable.

The fourth and final research question required evidence that the linear progression of the items on the Rasch item-person map was consistent with the construct theory and substantively meaningful. This map aids in making inferences about the characteristics of children from the constructed measure.

## 3. Results

### 3.1. Descriptive statistics

Basic descriptive statistics (M, SD) of the study’s major variables are presented in Table 1. Of the 127 respondents, the mean age was 12.59 (SD = 1.15, M_boy_ = 12.44, M_girl_ = 12.71). Our sample includes 58 boys and 69 girls (54.3%) (M = 0.54, SD = 0.5). Since the raw score total of ICQ scale were more frequently used by researchers, we also calculated the summary score of ICQ (M = 123.93, SD = 22.53). Specifically, boys reported higher ICQ summary scores than girls (M_boy_ = 127.92, M_girl_ = 120.56), but no statistically significant difference was found.

**Table 1:** Descriptive statistics.

Variable	Mean	SD	Min	Max	*p* value
Gender(% female)	0.54	0.5	0	1	
Age	12.59	1.15	9	14	
Language experience	1.22	0.41	1	2	
ICQ raw total	123.93	22.53	50	171	0.214
boys	127.92	22.67	73	171	
girls	120.56	22.2	50	157	

### 3.2. Reliability

The reliability results of the original ICQ scale are presented in Table 2 as “original.” Internal consistency reliability, as estimated from Cronbach’s alpha, was excellent at 0.91. The Rasch model analog to this with a person reliability of 0.92. The overall mean measure of interpersonal competence for the 127 children was +0.05 logits (SD = 0.56 logits), indicating the children, on average, found the ICQ items easy to endorse (mean item measure is conventionally set to 0). A person strata index was calculated in order to provide preliminary evidence of the external validity of the measure. In the present study, person separation was 3.41, which indicated that at least four measurably and arguably qualitatively distinguished strata of children on the ICQ variable, thus providing evidence of a quantifiable interpersonal competence measure. Model RMSE, the Rasch Model equivalent of the standard error of measure (SEM) in CTT, for children measure is 0.16, which is close to the REAL RMSE of 0.17, and indicates the relative precision in the model estimates of the children measures of interpersonal competence. The reliability for the 40 items was 0.90, meaning excellent reproducibility of the difficulty calibrations of the instrument. The item separation reliability value was 3.21, higher than the conventionally acceptable separation of 2.0, indicating that the items were functioning well and generated at least 3 quantitatively and qualitatively distinct groups of items.

**Table 2:** Summary of changes in separation, reliability, and variance explained.

Measure	Separation		Reliability		Variance explained
	Person	Item	Person	Item	
Original	3.10	3.07	0.91	0.90	32.7%
Option A	3.37	3.05	0.90	0.89	32.2%
Option B	3.33	3.20	0.90	0.90	35.0%
Option C	2.79	3.52	0.89	0.93	44.3%

Reliability and separation are expressed in logits. Reliability index of more than 0.80 indicate the most acceptable values (Costa and McCrae, 1992). Separation index exceeding 2.0 is considered good (Andrich, 1978), and the values less than 2 are weak (Giromini et al., 2015).

### 3.3. Validity

#### 3.3.1. Rasch principal component analysis

The initial dimensionality indices indicate that the measure only accounted for 32.7% of the person variance, less than half of the individual differences among these children’s responses. Since variance explained by measures is dominated by the variance of person abilities and item difficulties, it cannot confirm dimensionality by itself. Stronger indicators including “unexplained variance in the 1st and 2nd contrast” and item loading were therefore examined. The percentage of unexplained variance in 1st and 2nd contrast (8.4 and 7.4%, correspondingly) was higher than a conventionally accepted cutoff of 5.0%, which means there might be some systematic variance existing in the data that is unrelated to the original linear measure. The next step entailed the inspection of the item loading in the two contrasts to explore the presence of additional dimensions.

A factor loading of 0.5 was used as a conventionally accepted cutoff (Linacre, 2010). Items with the highest and the lowest loading were presented in Table 3. The first factor consists of responses to ICQ questions 10, 35, 5 and 20. Referring to the ICQ item contents, item 10 (Carrying on conversations with someone new whom you think you might like to get to know), item 35 (When angry with a companion, being able to accept that s/he has a valid point of view even if you do not agree with that view), 5 (Being able to admit that you might be wrong when a disagreement with a close companion begins to build into a serious fight), and 20 (Being able to take a companion’s perspective in a fight and really understand his or her point of view) express a common theme of conflict management. Items that had the lowest loading included item 11, item 21, item 26, and item 36. These items all described the ability to initiate relationships. Even though the percentage of variance was not large enough to create a measurement disturbance, it provided some evidence for the multidimensionality of the measure.

**Table 3:** Standardized factor loadings for principal component analysis of secondary factor.

Item	Factor loading
10. Being able to put begrudging (resentful) feelings aside when having a fight with a close companion	**0.69**
35. When angry with a companion, being able to accept that s/he has a valid point of view even if you do not agree with that view	**0.63**
5. Being able to admit that you might be wrong when a disagreement with a close companion begins to build into a serious fight	**0.59**
20. Being able to take a companion’s perspective in a fight and really understand his or her point of view	**0.53**
11. Carrying on conversations with someone new whom you think you might like to get to know.	**−0.6**
21. Introducing yourself to someone you might like to get to know.	**−0.6**
26. Calling (on the phone) a new date/acquaintance to set up a time to get together and do something	**−0.57**
36. Going to parties or gatherings where you do not know people well in order to start up new relationships	**−0.51**

Bold items indicate misfitting.

The unexplained variance in 2nd contrast was 7.4%, which is higher than a conventionally accepted cutoff of 5.0%. Items load onto this factor include item 39 (When a close companion needs help and support, being able to give advice in ways that are well received), 24 (Being a good and sensitive listener for a companion who is upset), 19 (Helping a close companion cope with family or roommate problems), 14 (Helping a close companion get to the heart of a problem s/he is experiencing), and 9 (Being able to patiently and sensitively listen to a companion “left off steam” about outside problems s/he is having) have higher loadings. These items share the same idea of providing emotional support, constitute a third component in the data. The results from Rasch principal components analysis indicated the possibility of multidimensions, as two potential factors were detected in the data.

#### 3.3.2. Item fit

Item statistics and item-measure correlations were then examined to evaluate the technical quality of items in the instrument. The fit statistics for all but one of the items were acceptable [an infit and outfit value within 0.60 to 1.40, Bond and Fox, 2015], indicating the items were generally measuring the same construct. However, item 12 (Turning down a request by a companion that is unreasonable) emerged as the outfit value was >1.4 (Bond and Fox, 2015), meaning this item indicated idiosyncratic variations from expectation. Items statistics are presented in Table 4, with misfitting items bolded.

**Table 4:** Items, fit, and item-total correlations for the original ICQ.

Item and number	Measure	Error	Infit MNSQ	Outfit MNSQ	Item-total correlation
Turning down a request (12)	−0.20	0.12	1.36	**1.56**	**0.06**
Revealing something intimate about yourself (3)	0.96	0.14	1.16	1.34	**0.17**
Not exploding at a close companion to avoid a damaging conflict (40)	−0.19	0.12	1.16	1.23	**0.20**
Confiding in a new friend about your softer, more sensitive side (8)	0.23	0.12	1.14	1.27	**0.27**
Refraining from saying things that cause a disagreement into fight (25)	−0.02	0.12	1.22	1.39	**0.28**
Being able to put begrudging (resentful) feelings aside (10)	0.17	0.12	0.93	0.93	**0.29**

Bold items indicate misfitting.

All items had positive item-measure correlations, most items with correlations between 0.30 to 0.70, meaning the item responses are in the direction of the latent variable. Many of the items have high item-measure correlations of above 0.50, meaning that higher ratings on these items were related to higher person measures. However, there were 6 items (items 12, 3, 40, 8, 25, 10) with small positive correlations (<0.30) (as shown in Table 4), which means that the items are either very easy or very difficult to endorse, or they may be functioning in a different way as the other items. Three of the misfitting items (item 40, item 25, and item 10) were also found to load onto the secondary factor identified in RPCA, which might signal problematic items not consistent with the construct (interpersonal skills).

#### 3.3.3. Person fit

Person fit statistics indicated that the sample performed well as expected in general. The three most misfitting respondents had both high infit and outfit statistics (>2.0), indicating unexpected responses to items both near to and far from their “ability” level. Note that the PT-measure correlation of misfitting respondent #26 was negative, and that of #50 was near zero. This might demonstrate that the individual score of these two respondents was contrary to those revealed in the sample’s overall response string.

#### 3.3.4. Rating scale functioning

Responses to the ICQ were expressed in a format of Poor, Fair, OK, Good, and Extremely Good, indicating the level of comfort in handling each of the situations described as questionnaire items. Codings for the responses were treated as 1, 2, 3, 4, and 5. According to Table 5, The reported mean-square values demonstrated that children’s use of the categories was productive for measurement (Infit MNSQ for category 4 is the lowest =0.90; Outfit MNSQ for category 5 is the highest =1.10). The observed average and Andrich threshold values both range from negative to positive, lowest to highest, however, the step difficulty between Poor/Fair threshold and Fair/OK threshold (0.31 logits) was far less than the generally recommended minimum of 1.4 logits. This means that students may had a problem distinguishing category 3 (I’m OK with it) with category 2 (I’m fair at this).

**Table 5:** Summary of category structure for the original ICQ measure.

Category Label	Average measure	Infit MNSQ	Outfit MNSQ	Step threshold	Step standard error
1	−2.22	1.03	1.05	None	
2	−0.89	0.91	0.9	−0.75	0.97
3	−0.04	0.98	0.99	−0.44	1.05
4	0.86	0.9	0.92	0.21	1.08
5	2.34	1.09	1.1	0.98	0.89

MNSQ, mean square; 1, Poor; 2, Fair; 3, Ok; 4, Good; 5 = Extremely Good.

**Figure 1:**
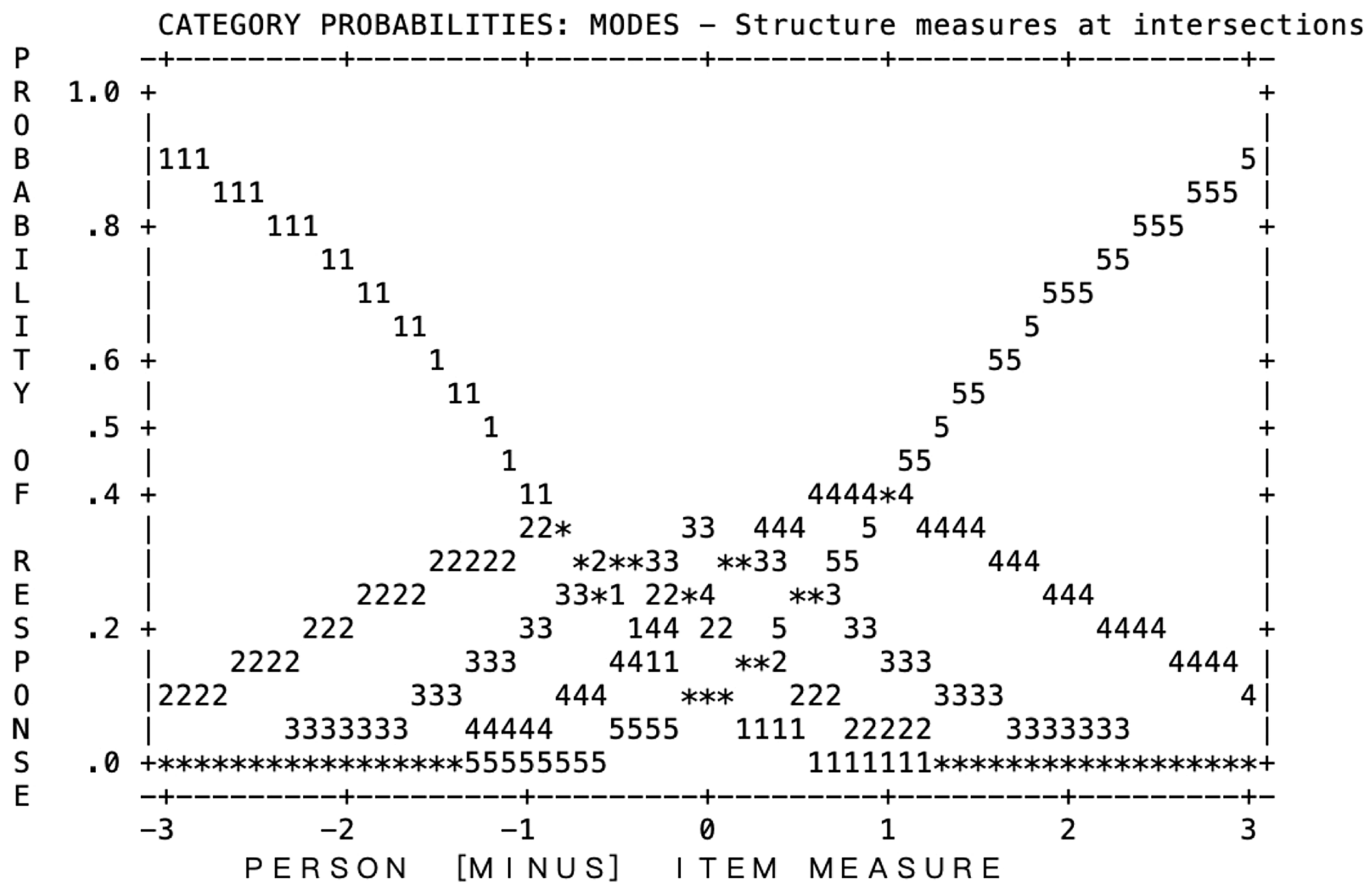
Category probabilities for original ICQ. 1, Poor; 2, Fair; 3, Ok, 4, Good; 5, Extremely Good.

The category probability curves (Figure 1) reinforced this confusion between categories 2 and 3, as shown in the Rasch modeled logistic ogives for the response category options (“1” for Poor, “2” for Fair, “3” for Ok, “4” for Good, and “5” for Extremely Good). According to Figure 1, when a child’s ability was the same as the item difficulty (person-item = zero), it is more likely for the child to endorse category “3” (OK) followed by category “4” (Good) and “2” (Fair). This is because category “3” (OK) has the highest probability of 0.4 (on the y-axis). Similarly, when the person-item difference is +3, the most likely response category is “5” (Extremely Good), followed by category “5” (Good). Because the threshold values for the highest probability peak for all categories are close to 0.4 (slightly less than the minimum of 0.5), this is further evidence that the category did not function as intended because students had difficulty distinguishing between “Fair” and “OK.”

### 3.4. Refinement of measure

Because the initial measurement analysis of the ICQ scale presented some problems with unidimensionality, confused rating scale categories, and several misfitting items, additional iterations were conducted to improve the fit of the data to the Rach model. Four items were flagged by the Principal Components Analysis and fit statistics. Fit statistics and item-measure correlation further identified several misfitting items. Upon further examination, items 10, 12, and 40 were the most problematic. Therefore, the first option (option A) to improve the measure was to delete these three items and collapse the two confused rating scale options. A second option (option B) included the deletion of all items that loaded onto the first factor and collapsing the two confused rating scale options. A third option (option C) was to delete all items that loaded onto the first factor (item 5, 10, 20 and 35) and those with low item-measure correlations (item 3, 8, 12, 25, and 40), remove misfitting people, then rerun the Rasch analysis with the new collapsed rating scale. Table 2 below summarized the statistics of the three options compared with the original measure including person and item separation statistics, reliability correlations for person and item, as well as total variance explained by the measure. Based on the statistics provided, option C was the most desirable, as the variance explained was improved to 44.3% without compromising the reliability. This new measure is referred to as ICQ-refined.

The results from Rasch ICQ-refined indicated that the measure was internally consistent and stable, and the person separation of 2.79 indicated that there were at least 4 statistically distinguishable groups of persons with various levels of interpersonal skills identified by ICQ. This was an adequate person strata, because the more strata the measure generated, the higher ability for the measure to cover the larger population. Further, the rating scale function was improved, with step thresholds spaced adequately and each category demonstrating sufficient fit. This means that each category reflected a meaningful distinction in interpersonal skills along the measured variable.

Item statistics for this new measure were also improved. All items had adequate item-measure correlations ranging from 0.32 to 0.67, meaning that the items’ responses were in the direction of the measured variable. The infit and outfit statistics for most of the items were adequate, only one item (13) had the infit and outfit value slightly higher than the conventionally accepted range. This improvement in item statistics provided further evidence for the content validity of ICQ-refined.

**Figure 2:**
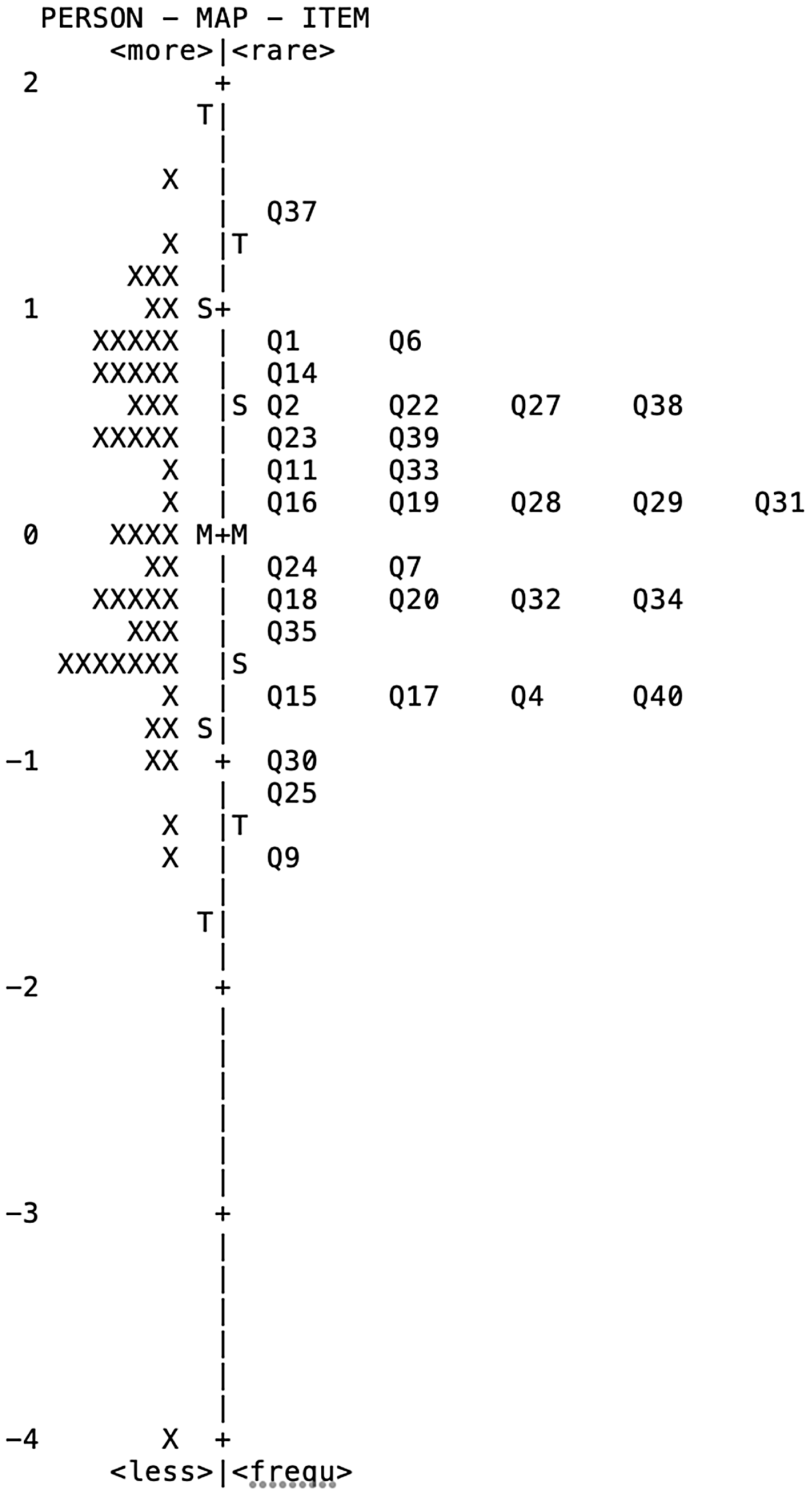
Person-item Map for ICQ-revised. The respondents are found on the left of the scale, with a greater number of high interpersonal skills respondents located at the top of the map. Items are presented on the right of the scale with items more difficult to endorse on the top. M, mean; S, 1 standard deviation (SD) from the mean; and T, 2 SD from the mean.

All persons had adequate infit and outfit value, and the point-measure correlation for persons was all positive, meaning that individual responses were consistent with the overall response string. The *item-person map* for ICQ-refined generated by Rasch analysis is displayed in Figure 2. The observed spread of item difficulties and person measures indicated that the measure generally covered the construct of interpersonal skills. The person measures were displayed in the left column, ranging from −3.99 to +1.57. The item measures were presented in the right column, ranging from −1.42 to +1.42. Additionally, the mean measure for person was 0.04, which was very close to zero. This provides evidence that the measure was well-targeted to this sample of children. However, by looking at the person-item map in Figure 2, there were more children than items located on the higher end of the distribution, meaning that there is a need to add items at the top end of the scale to measure those children who have high interpersonal skills.

According to Figure 2, the easiest items to endorse include those related to emotional support. For example, Q9 Being able to patiently and sensitively listen to a companion “left off steam” about outside problems s/he is having. Q24 Being a good and sensitive listener for a companion who is upset. Q29 Being able to say and do things to support a close companion when s/he is feeling down.Q39 When a close companion needs help and support, being able to give advice in ways that are well received, and Q4. Helping a close companion work through his or her thoughts and feelings about a major life decision, e.g., a career choice. The most difficult items to endorse include those related to starting a new relationship, disclosing personal feeling and conflict management. Items that are most difficult to endorse include the following: Q36 Going to parties or gatherings where you do not know people well in order to start new relationships. Q6 Finding and suggesting things to do with new people whom you find interesting and attractive. Q1 Asking or suggesting to someone new that you get together and do something, e.g., go out together, and Q13 Telling a close companion thing about yourself that you are ashamed of; To further elaborate, if we draw a line through the Keymap and centered at the average child response, we can see that the average child in this sample is most likely to respond “5” (I’m extremely good at this) to easier items (i.e., 9, 24, 29, and 39) and respond “2” to the most difficult ones including item 36 and 6, and response “4” to the average items, such as 33, 11, 28, 30, 15, and 18. The item hierarchy showed that the children who have higher interpersonal skills may display the characteristics of disclosing personal information, and the ability to initiate relationships with people they do not know. While students have lower interpersonal skills were more like to do better in providing emotional support than disclosing personal information or initiating relationships.

## 4. Discussion

This research attempted to validate a linear measure of interpersonal competence [developed by Buhrmester et al., 1988] that could be used among gifted children. Rasch RSM was used to obtain reliability and validity diagnostics for the ICQ scale. This is the first study to evaluate the psychometric properties of the ICQ scale in the gifted population using Rasch analysis. Although numerous studies have examined the reliability and validity of ICQ in U.S and Non-U.S. cultures (Kanning, 2006; Fisher, 2007; Górska, 2011; Giromini et al., 2015), we demonstrated how the Rasch model can provide unique information in addition to the Classical Test Theory commonly used by previous researchers, and empirically explained its application in measurement refinement.

In line with previous findings, the initial analyzes of the original ICQ scale produced satisfactory internal consistency reliability of 0.91. Rasch analysis demonstrated adequate overall reliability and good item fit for most of the items, but there was some misfit for item 12 with a misfit value that slightly exceeded the boundary of 1.40. This means that item 12 showed more observed variance than expected in the model. Regarding validity, the Rasch results also showed that the original ICQ scale was not functioning well as expected in terms of dimensionality, rating scale functioning, and fit statistics. Refinement was then made according to the initial results, including deleting problematic items and person and collapsing confused rating scale categories. Rasch analyzes provided support for the reliability, validity and functionality of this new ICQ-refined measure. This evidence also implied that interpersonal competence is quantifiable, which is an important step forward in understanding the complex construct of interpersonal competence as well as being able to measure it in a gifted population.

In the present study, we identified four distinct groups along the interpersonal competence continuum. Children with lower interpersonal competence were more likely to endorse statements referencing providing emotional support, while they were unwilling to initiate new relationships followed by disclosing their personal information to others. Statements about asserting displeasure with others and conflict management were relatively easy to endorse with our sample even for children with lower interpersonal competence. Such findings replicated the hierarchical structure presented in the construct theory of ICQ pretty well. Although the distribution of item difficulty is rather different, our results provided evidence that items related to initiating relationships and disclosing personal information are among the most difficult ones to endorse, while providing emotional support may need less interpersonal competence. Thus, the findings of the present study add to the understanding of the true meaning of interpersonal competence as a construct. For instance, it enabled us to identify which types of items, instead of those specific ones, elicit the most advanced interpersonal competence (initiating relationships and disclosing personal information), and which types of items elicit less advanced interpersonal competence (asserting displeasure with others and conflict management) as well as the least (providing emotional support). These findings, therefore, suggest that future discussion on the construct of interpersonal competence can move from a focus on specific items to the meaning of different levels of interpersonal competence.

The findings in this study yield several important contributions for the investment understanding the interpersonal skills of students who are gifted. First, the study contributes to current literature of measure development in the field of gifted education. Gifted students are different. Because of their heightened abilities, they usually experience more intense social and emotional problems. Interpersonal skills are important social and emotional characteristics that largely affected gifted students. However, there are no measures that were designed specifically for the gifted population. The measures for general population also have not been validated among gifted population. This study hereby begins the development of measures that can accurately and precisely measure interpersonal skills of the gifted children, thus helps to the better understanding the inner world of the gifted.

Second, the application of Rasch model in the development and validation of measures provide researcher and scholars a better understanding of the reliability and validity of measures. Psychometric analysis using traditional CTT examines internal consistency and convergent validity. However, IRT, specifically Rasch analysis, provides more information about a measure, such as external validity, structural validity, substantive validity, generalizability and so on (Wolf and Smith, 2007). This rich information can help researchers and scholars to better understand various aspects of validity and develop ways to improve the measure.

## 5. Conclusion

In conclusion, the study provided evidence that the construct of interpersonal skills is measurable. Based upon the original ICQ scale, the author extracted and piloted a refined measure that performed a meaningful, theoretically consistent linear progression measure that could be used to measure the level of interpersonal skills of gifted children. This ICQ-refined measure is a four-point measure consisting of 31 items extracted from the original ICQ. A strong set of reliability and validity evidence presented in the previous chapter supported the unidimensional nature of this refined measure. Therefore, this refined measure as ICQ-revised should be considered a better measure to be used for subsequent statistical analysis of gifted students’ interpersonal skills.

Future research will be continued to further investigate the interpersonal skills of gifted children. First, the overall theory of interpersonal skills should be sought for qualitative investigations, quantitative study based on larger sample size is also needed to provide more precise results. Further, with the help of better measurement tools, theoretical analysis is needed to design ways to improve interpersonal skills of gifted children. Well-designed field experiment should be conducted to evaluate the impact of such strategies on interpersonal skills of gifted children.

## Data availability statement

The raw data supporting the conclusions of this article will be made available by the authors, without undue reservation.

## Ethics statement

The studies involving humans were approved by Institutional Review Board of the University of Toledo (IRB number: 0000202422). The studies were conducted in accordance with the local legislation and institutional requirements. Written informed consent for participation in this study was provided by the participants’ legal guardians/next of kin.

## Author contributions

JY contributed to the concept or design of the study, collected and analyzed the data and draft the manuscript. ZR, YL, and SZ critically revised and formatted the manuscript. All authors contributed to the article and approved the submitted version.

## Funding

This research was funded by the 111 Project (Grant No. B16031), Soft Science Research Project of Xi’an Science and Technology Plan (Grant No. 23RKYJ0053).

## Conflict of interest

The authors declare that the research was conducted in the absence of any commercial or financial relationships that could be construed as a potential conflict of interest.

## Publisher’s note

All claims expressed in this article are solely those of the authors and do not necessarily represent those of their affiliated organizations, or those of the publisher, the editors and the reviewers. Any product that may be evaluated in this article, or claim that may be made by its manufacturer, is not guaranteed or endorsed by the publisher.
