# Supplementary material for: Applying Rasch analysis in refinement and validation of interpersonal skills measure for gifted children
Source: Front Psychol. 2023 Aug 31;14:1236640. doi: 10.3389/fpsyg.2023.1236640 (PMC10501403; doi:10.3389/fpsyg.2023.1236640)
Supplement: Supplementary file 1 [file Table_1.docx]

Appendix

Construct theory of the ICQ

| Items | Lables |
| --- | --- |
| 36 parties with someone you don’t know well  3 revealing something intimate to someone just getting to know  8 confiding in a new friend and let him/her see your softer, sensitive side  10 able to put resentful feelings aside when having a fight with a close companion  2 tell a companion you don’t how he/she treats you  17 standing up for your rights when a companion is being inconsiderate  32 telling a companion s/he hurts your feeling  37 telling a date/acquaintance that he/she made you angry | Initiating relationship  Disclosing personal information  Negative assertion |
| 18 letting a new companion get to know the “real you”  22 telling an acquaintance he/she embarrassed you  1 asking/suggesting to someone new to get together and do something  11 carrying on conversation with someone new  13 telling a close companion things you’re ashamed of  27 confronting your close companion when he/she broke a promise  26 calling a new date/acquaintance to set up an appointment  38 move a conversation beyond superficial talk  25 refraining from saying things that may cause a disagreement build into big fight  21 introducing yourself to someone you like to know  7 say no when asked to do things you don’t want  40 not exploding to avoid a damaging conflict  6 suggesting things to do with new people you find interesting  12 turning down an unreasonable request  33 telling a companion how much you appreciate and care for him/her  20 take a companion’s perspective in a fight  30 work through a problem with a companion without resorting to global accusations  35 accept other’s view even you don’t agree with that view | Conflict management |
| 19 helping a companion cope with family or roommate problems  23 trusting a close companion  34 show genuine empathetic concern even when the problem is uninteresting to you  28 telling a close companion that make you feel anxious or afraid  31 presenting good first impressions  15 listen to others’ complaints rather than “read” his/her mind when having a fight  4 help a companion work through thoughts about a life decision  16 being interesting and enjoyable when first getting to know people  5 able to admit you might be wrong when a disagreement begins to build into a fight  29 support a close companion when s/he is feeling down  39 give advice in ways that are well received  14 helping a close companion get to the heart of a problem  9 patiently and sensitively listened to a companion’s “left off steam”  24 a good and sensitive listener | Emotional support |
